# PeakAnalyzer: Genome-wide annotation of chromatin binding and modification loci

**DOI:** 10.1186/1471-2105-11-415

**Published:** 2010-08-06

**Authors:** Mali Salmon-Divon, Heidi Dvinge, Kairi Tammoja, Paul Bertone

**Affiliations:** 1 EMBL European Bioinformatics Institute, Wellcome Trust Genome Campus, Cambridge CB10 1SD, UK; 2 Department of Cell and Molecular Biology, Karolinska Institutet, S-171 77 Stockholm, Sweden

## Abstract

**Background:**

Functional genomic studies involving high-throughput sequencing and tiling array applications, such as ChIP-seq and ChIP-chip, generate large numbers of experimentally-derived signal peaks across the genome under study. In analyzing these loci to determine their potential regulatory functions, areas of signal enrichment must be considered relative to proximal genes and regulatory elements annotated throughout the target genome Regions of chromatin association by transcriptional regulators should be distinguished as individual binding sites in order to enhance downstream analyses, such as the identification of known and novel consensus motifs.

**Results:**

PeakAnalyzer is a set of high-performance utilities for the automated processing of experimentally-derived peak regions and annotation of genomic loci. The programs can accurately subdivide multimodal regions of signal enrichment into distinct subpeaks corresponding to binding sites or chromatin modifications, retrieve genomic sequences encompassing the computed subpeak summits, and identify positional features of interest such as intersection with exon/intron gene components, proximity to up- or downstream transcriptional start sites and *cis*-regulatory elements. The software can be configured to run either as a pipeline component for high-throughput analyses, or as a cross-platform desktop application with an intuitive user interface.

**Conclusions:**

PeakAnalyzer comprises a number of utilities essential for ChIP-seq and ChIP-chip data analysis. High-performance implementations are provided for Unix pipeline integration along with a GUI version for interactive use. Source code in C++ and Java is provided, as are native binaries for Linux, Mac OS X and Windows systems.

## Background

Next-generation sequencing technologies and tiling microarrays are frequently employed for genome-wide identification of regulatory elements and chromatin modifications. These applications generate vast numbers of experimental data points, which are compiled into extensive sets of genomic loci representing the units of biological activity measured in the particular assay. Researchers must then discern functionally-relevant results from these large-scale datasets, a process that poses significant bioinformatic challenges for research groups with limited computational support. For example, a common aim of transcription factor location analysis is to determine the relationship between ChIP-enriched loci and annotated genes; identifying the *cis*-regulatory elements occupied by the factor can reveal the set of genes it is likely to regulate across the genome. Correlating global transcription factor binding-site occupancy with target genes quickly becomes intractable in the absence of software tools to automate aspects of large-scale data analysis.

Sequence patterns occurring repeatedly among enriched loci are indicative of regulatory elements such as transcription factor-binding sites, and can often be identified by DNA motif analysis. Successful motif discovery relies on a set of candidate loci that exclude extraneous sequences while still containing the binding site consensus; however, since many peak-finding utilities merge overlapping areas of enrichment, the resulting peaks tend to be much larger than the actual binding sites. Peak regions often comprise more than one functional element (e.g. co-located transcription factor-binding sites or chromatin modifications), and these must be distinguished into individual loci in order to accurately interpret experimental results. The ability to subdivide composite peak regions into a finer-resolution set of individual binding sites (subpeaks) can improve the accuracy of sequence motif analysis.

Here we describe PeakAnalyzer, a set of standalone tools for the automated post-processing of large-scale chromatin profiling data. The programs are able to identify discrete enrichment peaks from loci corresponding to transcription factor binding or chromatin modification, retrieve individual peak sequences and annotate experimental data against various classes of functional elements, such as genes, CpG islands, regulatory features or DNase I hypersensitive sites. Results can also be compared across multiple datasets to report overlapping features, as well as those unique to a given experimental sample. The software is freely available and flexible in implementation, providing both high-performance solutions for pipeline integration and a GUI version for desktop users.

## Implementation

### Program description

**Figure 1:**
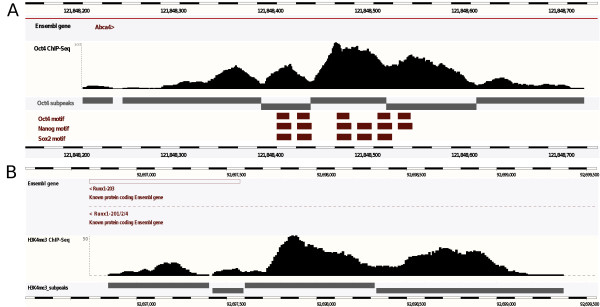
**Experimentally-derived peak regions subdivided into individual sites of signal enrichment**. A) Region of Oct4 binding in the *Abca4 *gene locus, subdivided into six individual loci by the *PeakSplitter *utility. The canonical binding sequences of Oct4, Nanog and Sox2 are concentrated in the center peaks, while potentially novel motifs or sites of associated co-factor binding may be present in the adjacent three. Peak splitting is equally applicable to the broader regions of enrichment generated from chromatin modification experiments, where signal peaks are more variable in size and shape. Depicted is a region of H3K4me3 methylation on chromosome 16 [29], subdivided into four discrete modification sites.

PeakAnalyzer comprises two main utilities: *PeakSplitter *and *PeakAnnotator. PeakSplitter *accurately subdivides experimentally-derived peak regions containing more than one site of signal enrichment, optionally retrieving genomic DNA sequences corresponding to subpeak summit regions. This procedure facilitates more detailed analysis of individual subpeaks (Figure 1). *PeakAnnotator *scans the target genome to identify and report functional elements proximal to peak loci and contains three main subroutines: Nearest Downstream Gene (NDG), Transcription Start Site (TSS) and Overlap Data Sets (ODS).

The function NDG locates the nearest downstream genes on both strands and calculates their distances. If the peak region intersects a gene, the program determines if the overlap is within an exon, intron, 5' UTR or 3' UTR. Multiple transcripts or genes overlapping a given location are all reported, providing a means to identify putative bi-directional promoters where the peak is proximal to genes on both strands. TSS locates the nearest transcriptional start site relative to each locus, scanning both downstream or upstream of the experimental peak to account for transcription initiation on either the sense or antisense strand. The ODS function calculates the overlap in positions/peaks between datasets, where peak loci intersecting by at least one nucleotide on either strand are reported. To compute a *P*-value of overlap enrichment, a random dataset is generated having peak lengths and chromosomal distribution matching the experimental dataset; the overlap between experimental and artificial loci is then determined, and through successive iterations a *P*-value representing the statistical significance of experimental signal over random is calculated.

### Software distribution and input requirements

PeakAnalyzer is implemented as a unified Java program encompassing the software components described above. Equivalent versions of *PeakSplitter *and *PeakAnnotator *are also implemented in C++ and Java so that users can choose a distribution suited to their particular requirements. Core facilities processing numerous datasets have the option to incorporate the faster C++ version into a Unix pipeline, whereas the Java implementations can either be run as separate command-line utilities or as a single cross-platform desktop application using an intuitive graphical interface.

PeakAnalyzer requires only a single peak file and a feature annotation file in BED or GTF format; complete annotation files for the current builds of the human (HG19) and mouse (MM9) genomes are provided with the software distribution. The input files required by *PeakSplitter *are those commonly generated by peak-finding programs: a .bed-formatted peak file containing chromosome start and end locations of signal enrichment loci, and a .wig signal file describing the size and shape of each peak.

### Algorithm implementation

#### PeakSplitter

We adopted the peak-splitting approach proposed by Fejes et al. [1] and implemented as the function subpeaks in recent versions of their FindPeaks tool. The method identifies multiple peaks within a given locus and accurately subdivides those containing more than one site of signal enrichment. In addition to incorporating the algorithm into PeakAnalyzer we provide a standalone version as the *PeakSplitter *utility, thereby enabling its application to signal loci called by any such program (e.g., [2-9]). Local maxima are identified in the peak region by scanning for relative peak heights, where those of adjacent maxima are compared and the lowest value is multiplied by a user-adjustable parameter to arrive at the read depth required for subpeak division. Binding sites are most likely to appear at or near subpeak summit regions, and these sequences can be retrieved directly from the Ensembl database [10].

#### PeakAnnotator

The *PeakAnnotator *component scans the target genome to identify and report functional elements proximal to peak loci. Rather than comparing each peak with all possible features, *PeakAnnotator *uses a combination of binary search and a modified version of the nested containment list (NCList) algorithm (see below and [11]) to rapidly identify proximal features among the full set of annotated elements. Proof of correctness of the algorithms described below and a discussion of their runtime complexity can be found in Additional file 1.

#### Generating a containment list

Determining the set of intersecting genomic regions across multiple experiments and data sources is not straightforward, because for a given dataset the regions queried may not be contiguous and some regions may be embedded within others. Thus, when sorting the regions by start position, the corresponding end positions could be out of sequence. This is more likely to be the case in higher eukaryotes where some loci encode overlapping genes.

The NCList algorithm constitutes a solution to this problem [11]. In this method the set of genomic regions is partitioned into a primary category of positionally-independent loci, and all remaining loci are segregated into a second category. We adopted this approach in our algorithm, where for each gene in the list *PeakAnnotator *creates a sublist of all genes containing it. A pseudocode description of the process is listed in Additional file 1, Figure S1.

#### Finding proximal downstream genes

The NDG utility determines the most proximal non-overlapping downstream genes on both strands. If a gene intersects a signal peak it will be stored in a separate list of overlapping genes. For simplicity, we define here a gene that is transcribed from the forward strand pos_gene, and a gene transcribed from the reverse strand neg_gene. The algorithm works as follows: the first non-overlapping gene located 3' to the peak, *G*_*3'*_, is found using a binary search strategy such that *G*_*3'-start *_>*Peak*_*end*_. If *G*_*3' *_is a pos_gene, it is the closest downsteam gene on the forward strand; if not, genes located downstream to *G*_*3' *_are visited until a pos_gene is found.

Next, the first gene located upstream to *G*_*3' *_that does not overlap with the current experimental peak is found, termed *G*_*5'*_. If *G*_*5' *_is a neg_gene it has the potential to be the closest downstream gene on the reverse strand. However, if *G*_*5' *_is contained within another gene transcribed from the reverse strand, this gene is potentially closer to or even intersecting the current peak. Hence, the next step is to determine the closest neg_gene and overlapping genes in the set of *G*_*5' *_and the gene(s) containing *G*_*5'*_. If *G*_*5' *_is a pos_gene, genes located upstream are visited until a neg_gene is found. Finally, the closest downstream neg_gene is searched within the set of that gene and those containing it.

#### Finding proximal transcription start sites

The TSS function works as follows: the first gene located downstream to the peak's central position, *G*_*3'*_, is found using a binary search strategy, and its distance to the current peak is calculated. Genes located downsteam to *G*_*3' *_are visited until a gene that starts downstream of the *G*_*3' *_locus is found. The gene having the lowest distance from the signal peak is then marked as the closest downstream gene. Next, the first gene upstream to *G*_*3'*_, termed *G*_*5'*_, whose end position <*G*_*5'-start *_(i.e., *G*_*5' *_= *G*_3'-1 _) is found. Its distance, and the distance of all genes that contain it, is calculated in order to find the nearest upstream gene. The one representing the minimal absolute distance to the peak among the set of proximal downstream and upstream genes will be reported.

#### Finding overlapping data sets

The ODS function operates on two sets of peaks, denoted here *S*_*1 *_and *S*_*2*_, and iterates over all loci in *S*_*1 *_to find those intersecting by at least one nucleotide with loci in *S*_*2*_. For each locus *Ln *in *S*_*1*_, the first non-overlapping peak from *S*_*2 *_located 3' to *L1*, termed *L2*_*3'*_, is found using a binary search strategy such that *L2*_*3'-start *_>*L1*_*end*_. The algorithm then searches upstream of *L2*_*3' *_to determine if any peak intersects *L1*, until the first locus in *S*_*2*_, termed *L2*_*5'*_, is found having coordinates outside the boundaries of *L1*. Peaks containing *L2*_*5' *_can potentially overlap *L1*, and are also considered.

## Results and Discussion

To illustrate typical applications of PeakAnalyzer, we analyzed the genome-wide binding profiles of a series of transcriptional regulators (Ctcf, E2f1, Esrrb, Klf4, c-Myc, n-Myc, Nanog, Oct4, Stat3, Smad1, Sox2, Suz12, Tcfcp2l1 and Zfx) in mouse embryonic stem (ES) cells, determined using the ChIP-seq method [12]. We obtained the primary data from the NCBI GEO database (series GSE11431), mapped the sequencing reads to the mouse genome assembly using the Bowtie alignment program [13], and detected significant peaks of signal enrichment with MACS [2]. Subsequent analyses were performed on the set of chromatin-binding regions from each of these re-processed ChIP-seq datasets.

### Identification and subdivision of signal peaks

**Figure 2:**
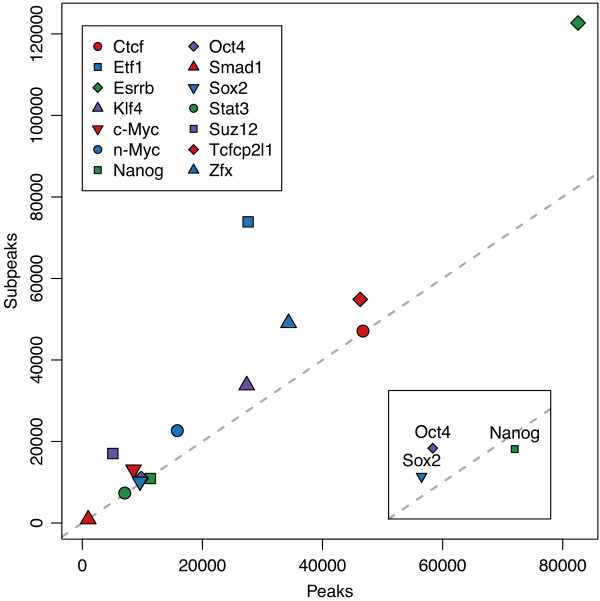
**Correlation between the total number of peaks and subpeaks for 13 transcription factors and the Polycomb group protein Suz12**. *PeakSplitter *was used to partition the regions of signal enrichment of each factor into individual binding loci. Significant agreement is observed between the numbers of putative binding sites resolved before and after processing (Pearson correlation = 0.93). Peak profiles corresponding to Oct4, Sox2 and Nanog are co-localized on the plot, indicating similar patterns of chromatin association across the genome.

In characterizing the binding patterns of each transcription factor, we first used the *PeakSplitter *utility to partition regions of signal enrichment into individual binding loci. The numbers of putative binding sites resolved for each factor before and after processing are summarized in Table 1. As illustrated in Figure 2, the number of original signal peaks roughly correlates with the number of subpeaks found by *PeakSplitter*. For some transcription factor proteins (Ctcf, Stat3, Nanog, Oct4 and Sox2), the total number of subpeaks is close to the original number identified; this suggests the presence of either a single regulatory element bound at each locus, or a small cluster of binding sites such that the combined distribution of peak regions is too uniform to be accurately partitioned. However, the binding profiles of Etf1 and Esrrb produced large numbers of additional subpeaks, where more than twice the original number of Etf1 binding sites were identified.

**Table 1:** Characteristics of transcription factor-bound peaks and subpeaks

Factor	Reads mapped	Peaks	Peak length	Subpeaks	Subpeak length	Motifs	
			(average/median)		(average/median)	peaks	subpeaks
Ctcf	3446024	46742	398/380	47117	332/319	94/94	95/95
Esrrb	11669746	82552	532/458	122689	315/299	135/166	215/246
Etf1	10245583	27612	1271/946	73888	441/390	-†	-
Klf4	6602662	27381	460/413	33781	301/289	45/55	65/68
c-Myc	10586180	8535	466/406	13115	263/249	0/18	12/27
n-Myc	7563562	15824	498/432	22688	284/269	0/32	16/46
Nanog	3201091	11334	412/385	10905	339/320	0/23	6/22
Oct4	7910224	9818	407/380	10928	293/289	8/20	20/22
Smad1	2530783	989	483/439	907	382/369	0/2	1/2
Sox2	8122529	9611	400/379	10159	316/309	14/20	21/21
Stat3	8533107	7069	326/293	7364	251/239	0/15	10/15
Suz12	8327215	5079	1550/1178	17043	430/389	-‡	-
Tcfcp2l1	10962390	46278	436/399	54856	324/310	2/93	102/110
Zfx	7323252	34348	486/406	49069	244/229	0/69	65/99

^† ^Alternate motif identified.

^‡ ^No consensus found.

Summary of ChIP-seq regions occupied by each transcription factor profiled, the numbers of peaks/subpeaks partitioned by *PeakSplitter*, and the datasets where previously reported binding motifs could be identified. The number of motifs present in transcription factor-bound peak and subpeak datasets are given in the last two columns, indicating improved motif detection from subpeak summit regions.

**Figure 3:**
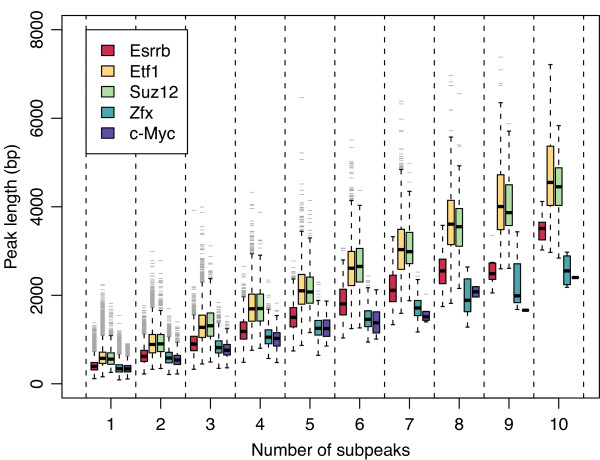
**Length of signal enrichment peaks relative to subpeaks derived from the same loci**. Five representative transcription factors are shown with ChIP-seq binding regions ranging from 9,000 (c-Myc) to 82,000 (Esrrb). These were subdivided into maximally 10 subpeaks per locus, and the lengths of the original regions plotted against the number of subpeaks identified.

A logical assumption when interpreting ChIP-seq data is that wider areas of signal enrichment may contain greater numbers of individual binding sites than narrow peak regions. To test this idea, we plotted the lengths of the original peaks resolved for each transcription factor relative to the numbers of subpeaks identified in each case by *PeakSplitter *(Figure 3). Broader peak areas were indeed subdivided into greater numbers of subpeaks, indicating the presence of composite binding loci. However, individual factors were found to exhibit varying length profiles within peak groups that were partitioned into the same numbers of subpeaks. For example, for a given number of subpeaks produced by *PeakSplitter*, Etf1 binding sites appear to be considerably longer than those of Zfx. This would indicate that the distance between co-localized DNA binding sequences specific to each transcription factors is different, an observation that may be related to the size of each transcription factor protein complex when co-factors are bound.

### Genome-wide annotation of transcription factor binding sites

Binding sites identified from ChIP-based experiments are usually categorized relative to genomic features, such as the frequency of binding to promoters, enhancers, gene structures or unannotated intergenic regions. Of primary interest in determining transcription factor targets is the location of binding sites relative to known transcriptional start sites. The relationship between promoter occupancy and differential gene expression can often identify genes directly regulated by a factor, but can also provide insight into the mechanisms by which it mediates transcriptional activation or repression. For example, factors that bind close to transcriptional start sites have been proposed to promote gene expression by stabilizing the association of general transcription factors at the core promoter elements; factors that bind to distal regions, either upstream or downstream of a gene locus, may regulate transcription by mediating, through a chromatin looping mechanism, the protein-protein contacts between distal complexes and the general transcriptional machinery bound at the promoter.

**Figure 4:**
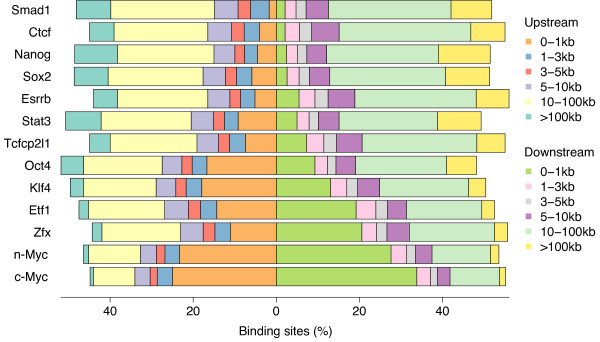
**Distribution of transcription factor-binding loci relative to 5' ends of genes**. The percentage of loci bound by each transcription factor downstream and upstream of all Ensembl transcripts was calculated using the TSS function of *PeakAnnotator*.

Here we used PeakAnalyzer to assign the genome-wide binding sites resolved for each of the 13 transcription factors to target genes, and profiled these interactions based on the distance between binding sites and gene loci. In [12], binding sites were assigned to target genes based on 17,762 annotated mouse promoters [14], which correspond to 17,442 non-redundant gene loci. Instead, we characterized the binding site profile of each factor separately in relation to all Ensembl-annotated transcripts. Using the TSS function in *PeakAnnotator*, we then calculated the percentage of binding sites downstream and upstream of Ensembl genes for each transcription factor profiled (Figure 4).

From this analysis, it appears evident that the binding profile of c-Myc is comparable with that of n-Myc, and that of Nanog is similar to both Sox2 and Smad1. Over 50% of c-Myc and n-Myc binding sites are located within or very close to target genes (up to 1 Kb), whereas only 25% correspond to distal binding sites (farther than 10 Kb). In contrast, distal binding sites constitute the predominant fraction (70%) of Sox2, Nanog and Smad1 loci; fewer than 10% of binding sites are found within or in close proximity to genes, suggesting that these factors bind preferentially to remote enhancer elements.

**Figure 5:**
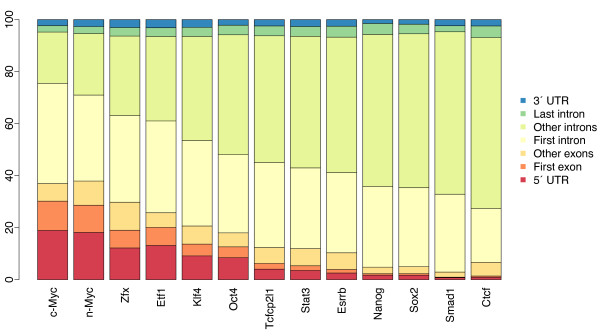
**Distribution of the central positions of individual transcription factor binding sites located within gene components**. Using the NDG function of *PeakAnnotator*, the percentage of transcription factor-binding sites falling within specific gene components was calculated. As illustrated here, some factors have a clear preference for promoters and regions of transcription initiation, while others exhibit a shift in binding-site occupancy from 5' regions to introns. Interestingly, the fraction of binding sites in last introns and 3' UTRs remain relatively constant among transcription factor-binding profiles, while the widest variation in within-gene composition occurs in the number of binding sites present in higher rank-order introns.

To further investigate the properties of binding sites located within genes, we used the NDG function of *PeakAnnotator *and plotted the percentage of sites that fall within different gene components (Figure 5). The within-gene composition of c-Myc binding sites approximates that of n-Myc, whereas the distribution of Nanog binding sites is most similar to Sox2. Both c-Myc/n-Myc occupy a large number of sites that fall within 5' UTRs and first introns (58%), whereas only 20% were found to intersect higher rank-order introns. In contrast, Nanog, Sox2 and Smad1 binding profiles are all characterized by a high percentage (60%) of sites within introns subsequent to the first, with sites intersecting the first intron comprising a lesser fraction (30%).

Unsurprisingly, c-Myc and n-Myc exhibit similar peak profiles, as Myc family members share gene and protein structural features [15] and function through common pathways [16-18]. Moreover, when expressed from the c-Myc locus, n-Myc is regulated in a similar fashion and functionally complementary to c-Myc in the context of various cellular growth and differentiation processes [19]. Although these two regulatory proteins display similar binding profiles, its not yet clear whether they share the same binding loci and regulate common target genes. To address this question we used *PeakAnnotator's *ODS utility to determine if c-Myc and n-Myc occupy the same binding loci in the ChIP-seq profiles examined. We found 7,039 (82%) of c-Myc binding sites to overlap those of n-Myc, with *P*-values < 0.001 compared to random peak locations. This observation indicates that, in the context of self-renewing ES cells, c-Myc and n-Myc are likely to participate in tandem to regulate the transcription of a large number of common target genes.

### Identification of regulated target genes

We next sought to correlate the number of peaks and subpeaks found either in the promoter regions of genes (up to 2 kb upstream) or within gene loci, relative to their corresponding expression levels in mouse ES cells. For this analysis, we obtained relevant microarray data from the GNF SymAtlas database [20], where expression levels from C57BL/6 mice were measured on the Affymetrix 430 2.0 array. Microarray probesets were mapped to 16,595 Ensembl-annotated genes, and these were subsequently partitioned into 7 gene sets based on log_2 _intensity values (from 2 to 16 in increments of 2).

**Figure 6:**
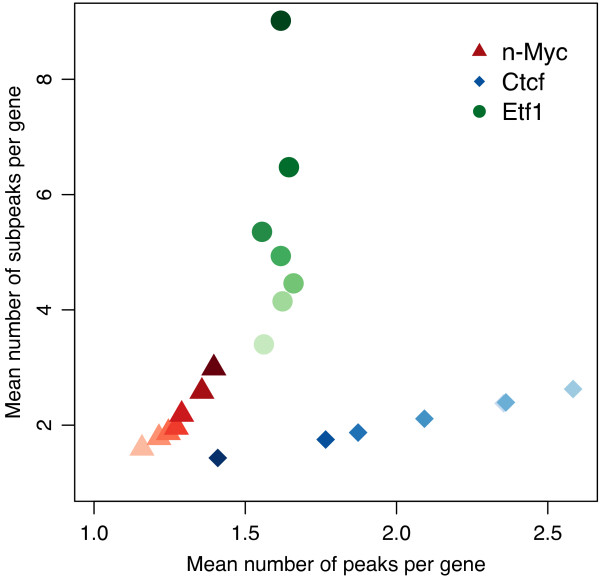
**Correlation between binding sites and target gene expression for three transcription factors**. The number of peaks and subpeaks identified in promoter-proximal regions (up to 2 Kb upstream) and within genes exhibiting different expression levels are plotted. Darker colors indicate higher expression.

Figure 6 illustrates the correlation between ChIP-seq binding sites and target gene expression for three (Etf1, n-Myc, Ctcf) of the 13 transcription factors. Positive correlation is observed between the numbers of n-Myc peaks and subpeaks relative to the abundance of regulated genes, where highly expressed genes have greater numbers of n-Myc binding sites. These can be identified in the signal peaks originally determined, and divided into a larger set of subpeaks by *PeakSplitter*.

In contrast, Ctcf occupies more binding sites in genes displaying lower expression levels, suggesting that in this context Ctcf acts as transcriptional repressor. Furthermore, the number of subpeaks resolved by *PeakSplitter *was much lower in this case, indicating the presence of single high-affinity Ctcf binding sites, possibly comprising several recognition sequences in close proximity. Interestingly, Etf1 occupies roughly the same number of loci per gene at all levels of expression, but these regions are split into significantly more subpeaks in highly expressed genes. This suggests that the frequency of binding to regulatory elements may enhance the expression of Etf1 target genes.

### Identification of binding motifs

A common aim in transcription factor-binding site analysis is to identify known and novel sequence patterns occurring within peak regions. To determine whether consensus binding sites are present in a set of ChIP DNA fragments, statistically over-represented subsequences can be found using motif discovery software. The accuracy of motif analysis relies on the specificity of the input sequences, as the presence of excessive flanking regions will often inhibit the detection of common patterns. It is therefore advantageous to reduce non-specific sequence content in order to minimize the amount of uninformative background from which motifs must be distinguished [21].

The ability to refine the set of input sequences can improve both the accuracy and success rate of motif discovery. In addition to subdividing signal peaks into discrete loci, *PeakSplitter *can be used to extract genomic DNA sequences corresponding to subpeak summit regions, which can then be used as input candidates for motif analysis. This feature is particularly useful when applied in conjunction with peak-calling software that does not report locations of greatest read depth within peak regions.

We employed MEME [22] to assess the performance of motif discovery using the subpeak summit sequences output by *PeakSplitter *relative to entire peak regions. The detection of new sequence motifs has been shown to plateau with a high number of input sequences [23]. Therefore we divided each ChIP-seq dataset into groups of 500 peaks, retrieved genomic DNA sequences corresponding to peak regions and used these as input to MEME. We then repeated this procedure using subpeak summit sequences as reported by *PeakSplitter*.

**Figure 7:**
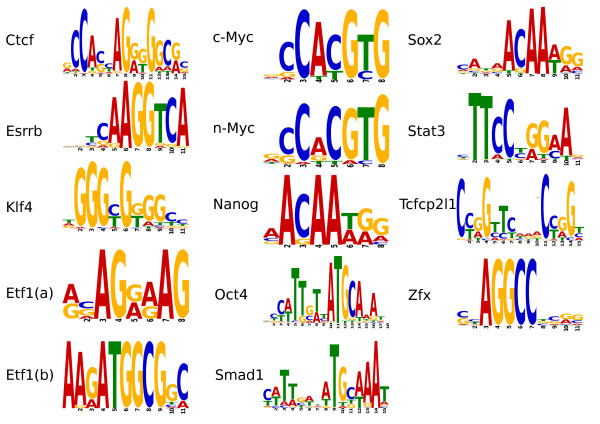
**Motif discovery from subpeak summit regions**. Identification of statistically over-represented sequences present in ChIP-seq binding loci, using the de novo motif discovery tool MEME [15]. The consensus sites are generally in agreement with those reported in [4], although in some cases (c-Myc, n-Myc, Nanog, Sox2 and Zfx) a shorter core motif was found. Two putative binding motifs have additionally been identified for Etf1, denoted here Etf1(a) and (b).

The number of peak/subpeak sets where a previously identified binding sequence for each transcription factor could be found are reported in Table 1. The consensus motif (Figure 7) was found for all factors using sequences corresponding to the summit regions reported by *PeakSplitter*, which was not the case when using entire peak sequences. Furthermore, processing sequences for motif discovery required significantly less computational time after applying *PeakSplitter*, on a high-performance compute cluster all 246 groups of 500 Esrrb subpeak summit sequences could be processed in under 3 hours, compared to 2.5 days to perform the same analysis on 166 full-length peak sets.

### Influence of peak-calling methods on motif discovery

The motif analysis described above could potentially be biased toward subpeak division if a particular peak-calling algorithm consistently reports longer peak regions than others. To verify whether this is the case, we compared the performance of *PeakSplitter *and subsequent motif discovery on the output of several alternative peak-calling utilities, using the Oct4 ChIP-seq profile as a representative example.

The sequencing data were first processed with six different peak callers: MACS [2], USeq [5], SISSRs [8], FindPeaks [1], ChIPSeqMini [6,24] and SWEMBL [25]. Default parameters were used in each case, with the exception of FindPeaks where a height threshold of 5 was applied to the output. The number of peaks reported by each peak caller is presented in Table 2, along with the peak length distribution. All peak callers except SISSRs report peak regions with median lengths between 261 (USeq) and 1189 (FindPeaks).

**Table 2:** Numbers of peaks and length distributions reported by various peak-calling programs

Program	Peaks	Length					
		min	Q1	Median	Mean	Q3	Max
FindPeaks ^†^	38837	301	821	1189	1379	1725	24970
SWEMBL	33475	100	296	381	429	494	8669
MACS ^‡^	9818	113	308	380	407	477	5983
ChIPSeqMini	4019	38	200	278	300	375	1291
SISSRs ^‡^	3498	40	40	60	82	100	640
USeq ^∓^	979	109	207	261	273	318	1521

^† ^Minimum peak height = 5

^‡ ^*P*-value < 1*e*^-5^

^∓^FDR 1%, fold change = 2

ChIP-seq data for Oct4 [12] were analyzed using six different peak callers. The numbers of peaks identified by each method along with the respective length distributions are listed.

We then applied *PeakSplitter *to subdivide the peak regions called by each program, and compared the number of subpeaks reported both with and without filtering based on minimum read depth. Such filtering is generally necessary to exclude spurious peaks in regions where sparse read mapping contributes to low-level background signal. The numbers of resulting subpeaks and their length distributions are listed in Table 3. The relative numbers of peaks differ significantly when the unprocessed .wig signal was used as input. Interestingly though, the peak length distributions are nearly identical across different methods.

**Table 3:** Numbers and length distributions of subpeaks

Program	Subpeaks	Length					
	unfiltered/filtered	min	Q1	median	mean	Q3	max
FindPeaks	414727/14177	4	41	51	60	65	607
Adjusted output	118724/45588	37	460	560	590	692	2490

SWEMBL	186472/13056	3	41	52	59	68	351
Adjusted output	88908/37095	2	145	185	195	233	1237

MACS	61283/8504	4	42	53	60	69	351
Adjusted output	16989/10928	39	220	289	293	359	1329

ChIPSeqMini	22704/7024	4	41	52	58	69	351

SISSRs	6854/2730	1	36	45	47	55	201

USeq	5100/2851	4	39	54	59	73	350

Experimental signal peaks output by different methods and subdivided using *PeakSplitter*. Subpeaks generated with and without filtering based on a minimal height threshold of 5 are reported, along with subpeak length distribution. Peak subdivision was applied based on a .wig file produced from the raw aligned reads, as only MACS, FindPeaks and SWEMBL output adjusted .wig files. For these methods *PeakSplitter *was also run using the modified .wig data generated by the respective program.

**Figure 8:**
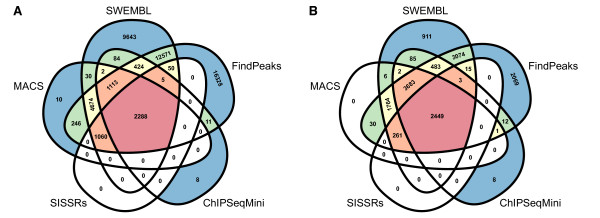
**Comparison of peak-calling methods**. Overlap of genomic regions identified by five different peak-calling algorithms, correlating the peaks (A) and subpeaks (B) generated from each on the Oct4 ChIP-seq dataset. The numbers of peaks/subpeaks shared between methods is similar, although FindPeaks and SWEMBL generated the highest numbers of unique calls.

We next examined the agreement between the output of each method by comparing the overlap between the reported peaks and subpeaks. A non-redundant list of peak loci was created by merging overlapping regions output by each program; the resulting numbers reflect how many called a peak within each site. The intersection is represented in Figure 8. FindPeaks and SWEMBL reported the highest numbers of peaks not supported by other methods, whereas USeq called the lowest number of peaks overall and is excluded from the figure for clarity. The relative overlap between the remaining five methods is similar when considering either the original peaks (Figure 8A) or subpeaks (8B).

Finally, we used these results to determine whether peak subdivision enhances motif discovery. The merged peak and subpeak datasets were divided into groups of 300 sequences and used as input to MEME. Since individual peak summit information is lost when regions called by different programs are merged, we used the entire peak sequences for motif analysis rather than regions flanking the summit. Following this analysis the canonical Oct4 binding sequence was not identified in any of the datasets containing the original peaks. After *PeakSplitter *was applied the motif was found in all of the subpeak datasets, aside from one instance where an Oct4 half-site was reported. These results indicate that subdividing signal peaks is essential for accurate motif discovery, independent of the original peak-calling method used.

## Conclusions

Regulatory elements identified through functional genomic assays are commonly determined based on signal peaks from tiling array fluorescence data or aligned reads from massively parallel sequencing. In order to interpret the results of such experiments, they must be considered in context with genes and regulatory elements in proximity to peak regions. Methods to automate the functional annotation of chromatin binding and modification loci can greatly ease characterization of their biological significance in genome-wide analyses.

A variety of tools are available for processing the primary data generated by ChIP-seq experiments, such as mapping sequence reads to a reference genome and identifying areas of significant enrichment. However, this is not the case for downstream analysis and data integration. Existing solutions that address these issues either rely on the transfer of large datasets via the Web for remote processing [26], require local installation of target genome databases [27], or operate within a specific computing environment [28].

PeakAnalyzer is a standalone solution amenable to a wide range of applications, including comparison of data generated on different experimental platforms. The software can accept any genomic loci as input and therefore can be used to process datasets spanning various methods, such as ChIP-seq, ChIP-chip, DamID, MeDIP and bisulfite sequencing. The *PeakAnnotator *component facilitates the automated annotation of numerous experimental results, and obviates the need to import large datasets into a genome browser for manual visualization and assessment.

Subdividing genomic loci with *PeakSplitter *is particularly useful for discerning individual binding sites that may be present in aggregate peak regions, and in extracting candidate sequences for motif analysis. We observe an increase in both accuracy and efficiency in motif search when ChIP data are processed by *PeakSplitter*. Partitioning broad signal peaks into discrete loci enriches the dataset for sequences containing transcription factor-binding sites and other regulatory elements, and can enhance the discovery of new consensus motifs by providing a more focused set of candidate sequences for alignment and/or model building.

## Availability and requirements

• Project name: PeakAnalyzer

• Project home page: http://www.bioinformatics.org/peakanalyzer or http://www.ebi.ac.uk/bertone/software

• Operating system(s): Platform independent

• Programming language: Java, C++

• Other requirements: Java 1.5 or higher, R for graphical output (optional)

• License: MIT/X Consortium

• Restrictions to use by non-academics: none

## Authors' contributions

MS-D and PB conceived and coordinated the study; MS-D developed the software with advice from PB; KT generated sample data for algorithm development; MS-D and HD analyzed the ChIP-seq data with advice from PB; MS-D, HD and PB drafted the manuscript and the content was approved by all authors.

## Supplementary Material

Additional file 1 **Supplemental material**. Algorithm proofs, procedural example of *PeakAnnotator *functionality, Figures S1 and S2, Table S1. Click here for file
