# Supplementary material for: PeakAnalyzer: Genome-wide annotation of chromatin binding and modification loci
Source: BMC Bioinformatics. 2010 Aug 6;11:415. doi: 10.1186/1471-2105-11-415 (PMC2923140; doi:10.1186/1471-2105-11-415)
Supplement: Additional file 1 — Supplemental material. Algorithm proofs, procedural example of PeakAnnotator functionality, Figures S1 and S2, Table S1. [file 1471-2105-11-415-S1.PDF]

Supplemental material

Salmon-Divon et al. (2010) PeakAnalyzer: Genome-wide annotation of chromatin binding and modification loci.

1. Proof of correctness for the algorithm to find the nearest downstream gene (`NDG`):

Let L be a genomic locus and G a vector of genes on the same chromosome. The vector is sorted according to the start position of each gene (i.e., if $i > j$ then $G[i]_{start} > G[j]_{start}$).

We would like to find:

1a) The closest gene on the forward strand located downstream to L.

For the forward strand case, a downstream gene D is defined as the gene having a chromosomal start position greater than the end position of L, i.e.

$$\min\left\{D_i = \left(G[i]_{start} - L_{end}\right)\right.$$

where $D > 0$ and $G[i]_{strand}$ = positive.

1b) The closest gene on the reverse strand located downstream to L.

For the reverse strand case, a downstream gene D is defined to be the gene having a chromosomal end position less than the start position of L, i.e.

$$\min\left\{D_i = \left(L_{start} - G[i]_{end}\right)\right.$$

where $D > 0$ and $G[i]_{strand}$ = negative.

Claim 1a:

Searching for a gene that satisfies the above criteria is equivalent to finding a minimal index i in $G[\mathrm{i}]_{start} > L_{end}$ where $G[i]_{strand}$ = positive, and taking the distance between $G[i]$ and L.

Proof:

Assume an index $j > i$, where $G[j]_{strand}$ = positive but $(G[j]_{start} - L_{end}) < (G[i]_{start} - L_{end})$.

$G[j]_{start} < G[i]_{start}$, but since the vector is sorted by start position, if $G[j]_{start} < G[i]_{start}$ then $j < i$, contradicting the assumption. ■

Claim 1b:

Searching for a gene that satisfies the above criteria is equivalent to finding a maximal index i in $G[i]_{end} < L_{start}$ where $G[i]_{strand}$ = negative, and taking the distance between $G[i]$ (or a gene containing $G[i]$) and L.

Proof:

Assume an index $j < i$, where $G[j]_{strand}$ = negative but $(L_{start} - G[j]_{end}) < (L_{start} - G[i]_{end})$.

$G[j]_{end} > G[i]_{end}$, but since the vector is sorted by start position and $j < i$, $G[j]$start < $G[i]$*start*;
$\therefore$ $G[j]$ must contain $G[i]$. ■

2. Proof of correctness for the algorithm to find the nearest transcription start site (TSS):

Let L be a genomic locus and G a vector of genes on the same chromosome. The vector is sorted according to the start position of each gene (i.e., if $i > j$ then $G[i]_{start} > G[j]_{start}$).

We would like to find the gene closest to L, where the distance is equal to the difference between the central point of locus L and a gene's transcriptional start site [i.e., `abs`($L_{center} - G_{tss}$)].

Claim 2a:

> Let t be the lowest index where $L_{center} < G[index]_{start}$, and let m be the index of the closest gene where $m \geq t$. It follows that $G[m]_{start} \leq G[t]_{end}$.

Proof:

> If $G[t]_{end} < G[m]_{start}$, it follows that $G[t]_{tss} < G[m]_{start}$ and that $G[t]_{tss} < G[m]_{tss}$. In this case the distance from L to $G[t]$, would be $d_t < d_m$, contradicting the original assumption. ■

Claim 2b:

> Let t be the lowest index where $L_{center} < G[index]_{start}$; $G[t\text{-}1]_{start} < L_{center}$. Let t' be the highest index where $G[t']_{end} < G[t\text{-}1]_{start}$, and m' the index of the closest gene where $m' < t$; hence $m' > t'$ or $G[m']$ contains $G[t']$.

Proof:

> As stated above, $G[t\text{-}1]_{start} < L_{center}$. Assume $m' \leq t'$ and that $G[m']$ does not contain $G[t']$. It follows that $G[m']_{start} \leq G[t']_{start}$, and $G[m']_{end} < G[t']_{end}$ (otherwise it would contain $G[t']$). In that case, $G[m']_{tss} < G[m']_{end} < G[t\text{-}1]_{start} < G[t\text{-}1]_{tss}$. Therefore t-1 would be closer than m' to L, in contradiction to the stated conditions. ■

3. Proof of correctness for the algorithm to determine the overlap between datasets (`ODS`):

Let L1 and L2 be two sets of genomic loci on the same chromosome. We wish to mark all loci in L1 having genomic coordinates intersecting those in L2, and vice versa.

Claim 3a:

> If the boundaries of locus A intersect locus B, then reciprocally B intersects A.

Let L1[x] be a locus in the set L1. We wish to find all loci in L2 intersecting the boundaries of L1[x].

Claim 3b:

> Let t be the lowest index where $L1[x]_{end} < L2[index]_{start}$, and t' the highest index $t' < t$ and L2[t'] does not intersect L1[x]. If m is a locus overlapping L1[x], then $t' \leq m < t'$, or L2[m] contains L2[t'].

Proof:

> Assume that $t \leq m$; $L1[x]_{end} < L2[t]_{start} \leq L2[m]_{start}$. Therefore L1[$x$] and L2[$m$] could not overlap, in contradiction to the conditions set forth above. Assume further that $m < t'$, and that L2[m] does not contain L2[t']. Therefore $L2[m]_{start} < L2[t']_{start}$. $L2[m]_{end} < L2[t']_{end}$ (otherwise L2[m] would contain L2[t']), but $L2[t']_{end} < L1[x]_{start}$. In that case $L2[m]_{end} < L1[x]_{start}$ and L1[x] and L2[m] cannot overlap, contradicting the original assumption. ■

```
Let x := a list of genes,
    S := a stack of contained genes,
    Gi := a set of genes containing gene i

for(i = 0, i < x_size, i++)
    if(x[i] ⊃ x[i + 1])
        S ← push x[i]
    else
        for each e in S (LIFO)
            if(x[i + 1] ⊄ e)
                S ← pop e
    Gi ← S
```

Figure S1. Pseudocode for creating a containment list

Runtime complexity

The procedure for generating a containment list object ($O(n)$) is outlined in Figure S1.

In the general case, the data structure is traversed using binary search to find the lowest-index element $G_{3'}$ with start position > the end position of a peak locus. Since the vector of input genes is sorted according to start position, the runtime complexity is $O(\log n)$.

The nearest downstream gene (`NDG`) on the forward strand, as described in claim and proof 1a, is found by traversing the vector in increasing order from $G_{3'}$ until a positive-strand gene is visited. `NDG` on the reverse strand is found by traversing the vector in decreasing order from $G_{3'}$ until a negative-strand gene which does not overlap the locus is visited. This gene, termed $G_{5'}$, or one of the genes containing it, is determined to be the nearest downstream reverse-strand gene according to claim and proof 1b.

Both are typically constant-time operations, averaging $O(\log n)$ and $O(n)$ in the worst case. For all peak loci the runtime complexity is $O(n \log n)$, and $O(n^2)$ in the worst case.

Procedural example of PeakAnnotator functionality

To illustrate the program's operation, we consider a genomic interval located at positions 500-6000 on a hypothetical chromosome. This region contains six genes as indicated in Figure S2, and a single peak locus. G5 is the closest downstream gene to the peak on the forward strand, and G1 is the closest downstream gene on the reverse strand. The boundaries of G3 intersect the peak. Whereas closest downstream genes are found in relation to a peak's start/end position, the distance is calculated between the central position of a peak and the 5′-end of a gene.

The algorithm proceeds as follows: G4 is identified as the closest non-overlapping gene located 3′ to the peak, where its start position (4500) > the peak's end position (4000). G4 becomes $G_{3'}$ of the general case (see *Algorithm implementation*). G4 is transcribed from the reverse strand, and is therefore located upstream to the peak. Hence the algorithm continues to visit genes downstream to G4 until a positive-strand gene (`pos_gene`) is found. In this example the closest downstream gene on the forward strand is G5.

The next step is to determine whether the gene located upstream to $G_{3'}$ intersects the peak, and continue to do so until a non-overlapping gene is found. This gene is termed $G_{5'}$. In this example, G3 is located upstream to G4. G3 intersects the peak locus, and will be stored in a list of overlapping genes. The next upstream gene is G2 (rather than G1, since this is determined according to start position), where G2 does not intersect the peak. G2 is oriented on the reverse strand, so it may be the closest downstream `neg_gene`. However, G2 is also contained within another gene transcribed from the reverse strand: G1. G1 is therefore reported as the closest downstream gene on the reverse strand.

In order to find the closest transcriptional start site (TSS) to an experimental peak, the distance to each TSS from the peak is calculated (Table S1). For clarity we assume that each TSS is equal to the gene's 5′-end. The algorithm first locates G4, which is the first gene downstream of the peak's central position. G4 is transcribed from the reverse strand, so its end position is annotated as the TSS, and other downstream genes may have a TSS closer to the peak locus. Hence, the algorithm searches downstream to G4 until a gene is reached that starts after G4 ends. In this example, G5 and G6 are located downstream to G4 and start before G4 ends, hence their distances are compared to find the minimal one, in this case G5.

The algorithm then searches upstream of G4 ($G_{3'}$ in the general case) until a gene is found having an end position < the start position of $G_{3'-1}$. In this case, G3 is the closest gene upstream to G4. G2 is the first gene having an end position < the start position of G3, and hence the minimal distance will be searched among all genes from G3 to G2, including those genes that contain G2. The closest TSS upstream to the peak locus is G1, where the distance (750bp) is less than the distance of the closest downstream gene (G5 = 950bp).

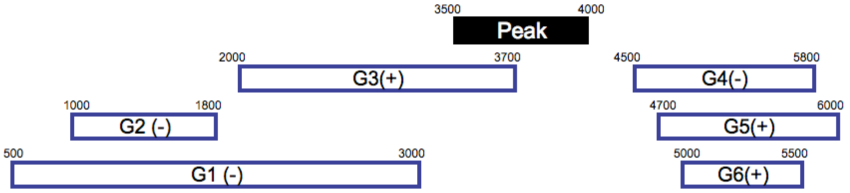

Figure S2. Hypothetical genomic region containing one binding site/signal peak and six adjacent genes. Signs (+/-) indicate strand orientation.

Gene	Strand	TSS	Distance to peak central position (3750)
G1	-	3000	3000-3750 = -750
G2	-	2700	1800-3750 = -1950
G3	+	2000	3750-2000 = 1750
G4	-	5800	5800-3750 = 2050
G5	+	4700	3750-4700 = -950
G6	+	5000	3750-5000 = -1250

Table S1. Distances of transcription start sites relative to the peak depicted in Figure S2 above.
